# The Topology of the Leg Joints of the Beetle *Pachnoda marginata* (Scarabaeidae, Cetoniinae) and Its Implication for the Tribological Properties

**DOI:** 10.3390/biomimetics3020012

**Published:** 2018-06-08

**Authors:** Steffen Vagts, Josef Schlattmann, Alexander Kovalev, Stanislav N. Gorb

**Affiliations:** 1 Department of System Technologies and Engineering Design Methodology, Hamburg University of Technology, Denickestr. 22, D-21079 Hamburg, Germany; j.schlattmann@tuhh.de; 2 Department of Functional Morphology and Biomechanics, Kiel University, Am Botanischen Garten 9, D-24118 Kiel, Germany; akovalev@zoologie.uni-kiel.de (A.K.); sgorb@zoologie.uni-kiel.de (S.N.G.)

**Keywords:** locomotion, walking, leg, joints, topology, insect, Arthropoda, friction, contact mechanics, biotribology

## Abstract

Locomotion of walking insects is exceptionally efficient. The function of their leg joints in different movement scenarios depends on their kinematics and contacting conditions between moving parts. The kinematics was previously studied in some insects, but contact mechanics within the joints remains largely unknown. In order to understand the complex topology of the contacting surfaces of the leg joints in the Congo rose beetle *Pachnoda marginata peregrina* (Scarabaeidae, Cetoniinae), we have investigated the shape, the waviness, and the roughness of the joint base and its counter body by applying confocal laser scanning microscopy and white light interferometry. Additionally, we performed nanoindentation tests on the contacting joint surfaces, in order to analyze material properties (elasticity modulus and hardness) of the joint cuticle. We found two topological design principles of the contact surfaces that might be considered as adaptations for reducing frictional drag during leg movements. First, the contact pairs of all leg joints studied consist of convex and concave counterparts. Second, there is a smooth and a rough surface in contact in which microprotuberances are present on the rough surface. These principles might be potentially interesting for technical implications, to design bioinspired joints with both reduced friction and wear rate.

## 1. Introduction

For successful locomotion, living organisms need a combination of maximum friction required for acceleration, deceleration, and manoeuvring, combined with minimum friction in joints for economic energy expenditure [1]. Vertebrate bones that are joined with each other are covered by cartilage, which is the gliding surface of the joint. This system is rather well studied in extensive biomedical and biotribological literature [1,2]. The coefficient of friction within the vertebrate joints is very low (0.0026) [3], because the cartilage is porous and its material is filled with synovial fluid [4,5] that provides lubrication of contacting surfaces [6] and serves as a kind of damper under dynamic loads. Interestingly, the surface of cartilage is not ideally smooth. Its roughness can influence resulting friction. A roughness of 1 μm is typical of fetal cartilage, and 2.7 μm in healthy adults [7,8]. 

**Figure 1:**
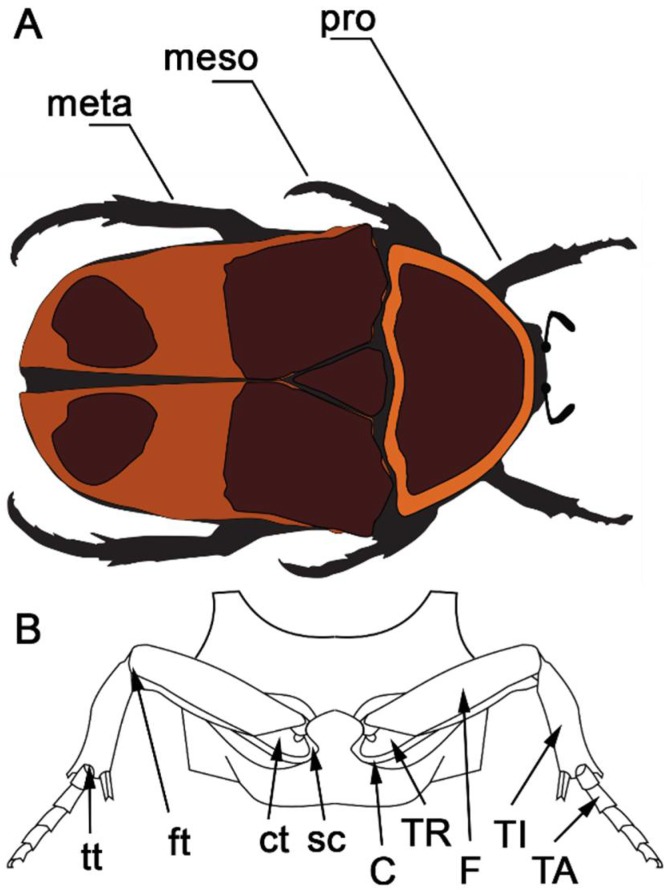
Schematic view of (**A**) the legs and (**B**) both the leg joints and segments of *Pachnoda marginata*. pro: prothoracic leg; meso: mesothoracic leg; meta: metathoracic leg; C: coxa; TR: trochanter; F: femur; TI: tibia; TA: tarsus; sc: subcoxa; ct: coxa–trochanter; ft: femur–tibia; tt: tibia–tarsus joint.

The structure of arthropod extremities is completely different from that of vertebrates (Figure 1). In insect leg joints, there are frictional surfaces that contact apparently under dry conditions and serve to prevent segment motion in certain directions or even fix it completely, in order to save muscle energy [9]. With regard to liquid lubricants, insect joints do not possess synovial fluid well known from the vertebrate joints [10]. Experimental studies on friction in insect joints have long been left open in the literature. But it has already been shown that the head joint surface of *Pachnoda marginata* is usually very smooth [11], but there are also rough surfaces, which presumably serve to prevent motion in one direction [1,9], in the joints of some beetles. The investigations of Kheireddin et al. [12] have recently shown that there are microstructural adaptations in the femoral–tibial joint of the lubber grasshopper (*Romalea guttata*), which may be responsible for the friction reduction in the joint.. Furthermore, the head–thorax joint of scarabaeid beetles shows that the contact pair consists of a harder convex and a softer concave surface [13]. Moreover, in the cetonine rose chafer *P. marginata*, the prothoracic part of this joint is rough and covered by a regular pattern of microstructures, whereas the opposed gula plate of the head is smooth [11,13]. 

The possible importance of microstructures in insect tribological systems has already been extensively discussed [14], but the amount of solid experimental data is very limited, especially in the case of the leg joints. Interestingly, these biological tribosystems can bear smooth surfaces or show specific microstructures on the surfaces of the joint [9]. Other organisms, such as reptiles, also have remarkable tribological properties on their surfaces that support their movement on various substrates. In the case of the ventral scales of snakes, for example, a friction minimizing effect, as well as direction-dependent friction behavior, have been demonstrated [15,16]. 

From the extensive tribological literature, we know that surface topography may strongly reduce friction. Suh et al. showed that undulated surfaces help to reduce the coefficient of friction of sliding bearings in comparison to smooth surfaces under dry conditions [17], as well as under the usage of solid lubricants such as molybdenum disulfide [18]. Moreover, Etsion et al. found that surface texturing with regular microdimples improves the frictional wear under dry [19], mixed friction, and starved lubrication [20]. These findings were successfully applied to thrust bearings [21] and piston rings [22].

Based on the previous data on the surface topography and mechanical properties of insect joints, we formulated two hypotheses about the role of the topology of contacting surfaces in insect leg joints for the reduction of friction: (1) The contact pair consists of convex and concave geometries, which fit each other well; and (2) there is always a smooth and a rough surface in contact on both corresponding counterparts of the joint. In order to understand the complex topology of the contacting surfaces of the leg joints in the Congo rose beetle *Pachnoda marginata peregrina* (Scarabaeidae, Cetoniinae), we have investigated the shape, the waviness, and the roughness of the joint base and its counter body by applying confocal laser scanning microscopy and white light interferometry. The obtained data might be potentially interesting for technical implications, to design bioinspired joints with both reduced friction and wear rate.

## 2. Materials and Methods

After analyzing the kinematic properties of legs in the beetle *P. marginata* [23], the identification of the contacting surfaces follows from the simulation data of the multibody system. The next deeper hierarchical view of this tribological system comprises the investigation and description of the two-dimensional (2D) and three-dimensional (3D) topologies of the articulation surfaces. In addition, further geometric data were obtained from the surfaces, which help in the further tribological analysis of the system and in the understanding of the contact mechanical interactions of the tribological system, including roughness effects [24].

### 2.1 Sample Preparation

The leg joints of *P. marginata* (*N* = 5) were conserved in alcohol. The beetles were taken from a colony at Kiel University. For the preparation, the relevant leg segment was first separated with scissors. The respective joint was opened with a scalpel and the two leg segments were separated. Subsequently, the surfaces to be examined were exposed, separated, and fixed on an object carrier using an adhesive (5925 Elastomer, Ergo, Kissing, Switzerland). For nanoindentation measurements, the beetle leg joint segments were prepared right before the experiment.

### 2.2 Confocal Laser Scanning Microscopy and White Light Interferometry

The traditional way to measure linear topology properties (tactile profilometry) is not suitable for the natural contact surfaces in the micrometer range because of some limitations like the destruction of soft surfaces, long measuring times, and critical calibration procedures [1]. Instead, optical methods, such as confocal laser scanning microscopy (CLSM) and white-light interferometry (WLI), are used to perform contactless measurements. These methods are best suited for the characterization of the surface texture, in order to analyze the waviness and roughness, as well as the primary profile, of the joint surfaces [25]. For understanding the tribological behavior, the information about the hierarchy (waviness and roughness) of two contacting surfaces is of great importance. It is to be expected that some surfaces have a microstructure that is difficult to quantitatively describe with the optical measuring methods. In this case, the structures are qualitatively described by scanning electron microscopy (SEM) investigations. In the present study, based on the preliminary SEM data, the surfaces of the beetle joints were analyzed using a Keyence VK-8710 confocal laser scanning microscope (Keyence Corporation, Osaka, Japan). Two brightfield objectives (Nikon CF IC EPI Plan Achromat 20×, numerical aperture = 0.46, working distance = 3.1 mm; Nikon CF IC EPI Plan Achromat 50×, numerical aperture = 0.8, working distance = 0.54 mm) were applied. The preparation was exposed to light from a stable solid-state laser with a wavelength of 635 nm (5 mW at the fiber end, laser power = 2%), and the laser light reflected at the surface of the joint surfaces was detected. The detector gain was automatically adjusted prior to image stack collection in a way that resulted in a maximum signal intensity with the simultaneous prevention of oversaturation. The image size was set to 1024 × 1024 pixels and the scan step size was 0.5 μm for 20× magnification and 0.1 μm for 50× magnification. The software VK-analyzer was used to create maximum intensity projections and color-coded height maps. Based on the results, the surface structures and radii of the joint surfaces were measured. The sample surface waviness and roughness were studied using a white-light interferometer ZygoNewView 6 K (Zygo Corporation, Middlefield, CT, USA). This technique can be used to obtain the average surface roughness (*R_a_*).

### 2.3 Nanoindentation

For further understanding the leg joint tribological system in *P. marginata*, the material properties (elastic modulus and hardness) of the contacting surfaces were measured by nanoindentation. The Nano Indenter SA2 (MTS Nano Instruments, Oak Ridge, TN, USA) equipped with a Berkovich indenter was used. The experiments were performed in continuous stiffness mode (CSM, 75 Hz) up to a 3 µm indentation depth. The calculation of elastic modulus and hardness was realized according to Pharr and Oliver [26] and Fisher-Cripps [27]. The Young´s modulus is labeled *E* and the hardness *H*.

### 2.4 Statistical Analysis

The statistical analysis of measurement data was performed with IBM SPSS Statistics (Version 22, Armonk, NY, USA). Analysis of variance was computed using the Kruskal–Wallis test by ranks.

## 3. Results

**Figure 2:**
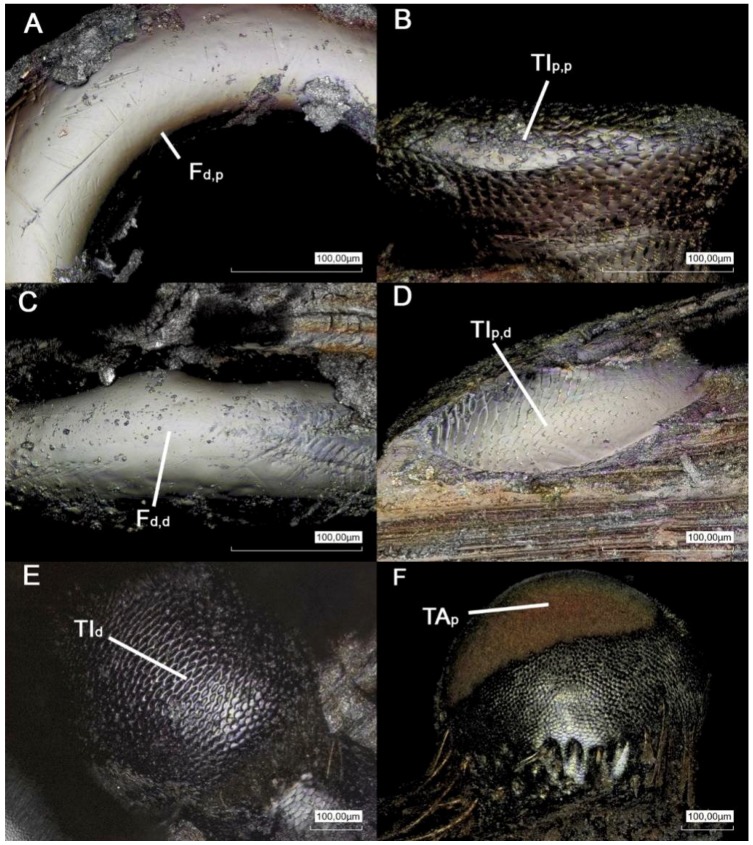
Confocal laser scanning microscopy images of the contacting surfaces in (**A**–**D**) the femur–tibia and (**E**,**F**) the tibia–tarsus leg joints of *P. marginata*. F_d,p_: the distal end of the femur, the proximal condyle; TI_p,p_: the proximal end of the tibia, the proximal condyle; F_d,d_: the distal end of the femur, the distal condyle; TI_p,d_: the proximal end of the tibia, the distal condyle; TI_d_: the distal end and the condyle of the tibia; TA_p_: the proximal end and the condyle of the tarsus.

**Figure 3:**
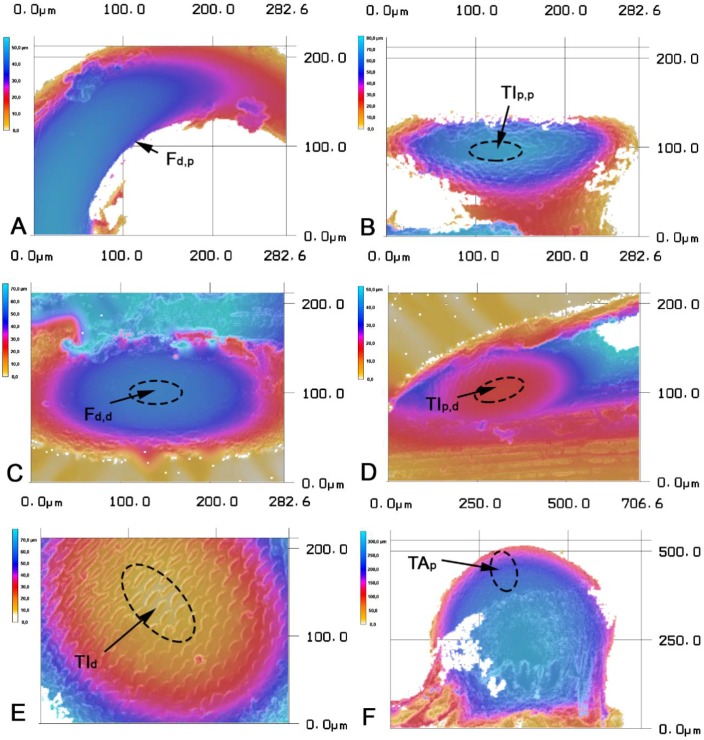
Confocal laser scanning microscopy rainbow-colored height profile images of the contacting surfaces in (**A**–**D**) the femur–tibia and (**E**,**F**) the tibia–tarsus leg joints of *P. marginata*. Dashed ellipses indicate the position of the condyle. F_d,p_: the distal end of the femur, the proximal condyle; TI_p,p_: the proximal end of the tibia, the proximal condyle; F_d,d_: the distal end of the femur, the distal condyle; TI_p,d_: the proximal end of the tibia, the distal condyle; TI_d_: the distal end and the condyle of the tibia; TA_p_: the proximal end and the condyle of the tarsus.

**Figure 4:**
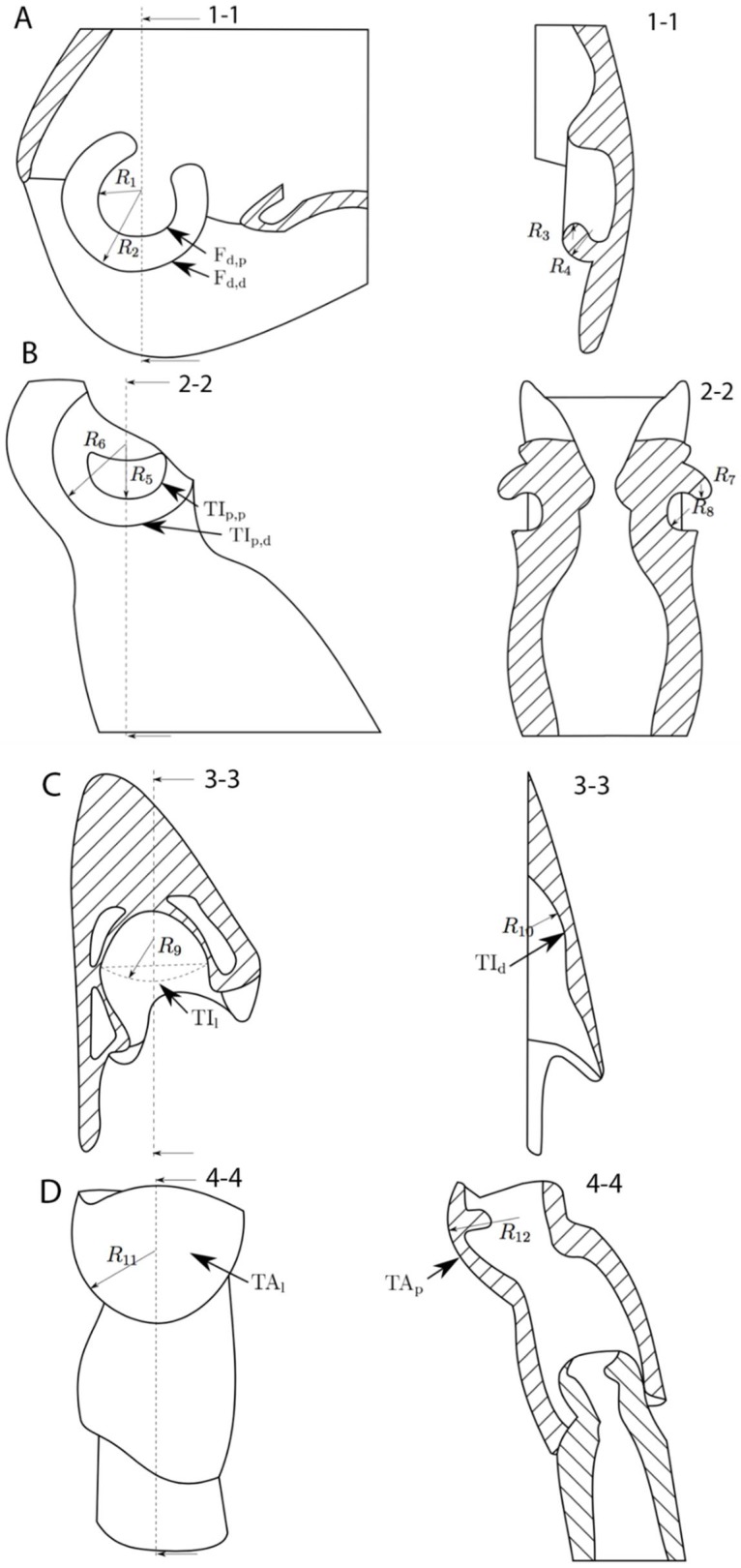
Schematic of the geometry of (**A**,**B**) the femur–tibia and (**C**,**D**) the tibia–tarsus leg joints of *P. marginata*. These schemes are based on three-dimensional analyses using confocal laser scanning microscopy (CLSM) and microcomputer tomography (µCT). The dashed lines show the position of section view. The filled areas represent the cross section of cuticle. F_d,p_: the distal end of the femur, the proximal condyle; TI_p,p_: the proximal end of the tibia, the proximal condyle; F_d,d_: the distal end of the femur, the distal condyle; TI_p,d_: the proximal end of the tibia, the distal condyle; TI_d_: the distal end and the condyle of the tibia; TA_p_: the proximal end and the condyle of the tarsus; TA_l_: the lateral curvature of the tarsus condyle; *R*_1–12_: radii of contacting joint surfaces; 1-1 to 4-4: sectional views of the geometry of the joints.

**Figure 5:**
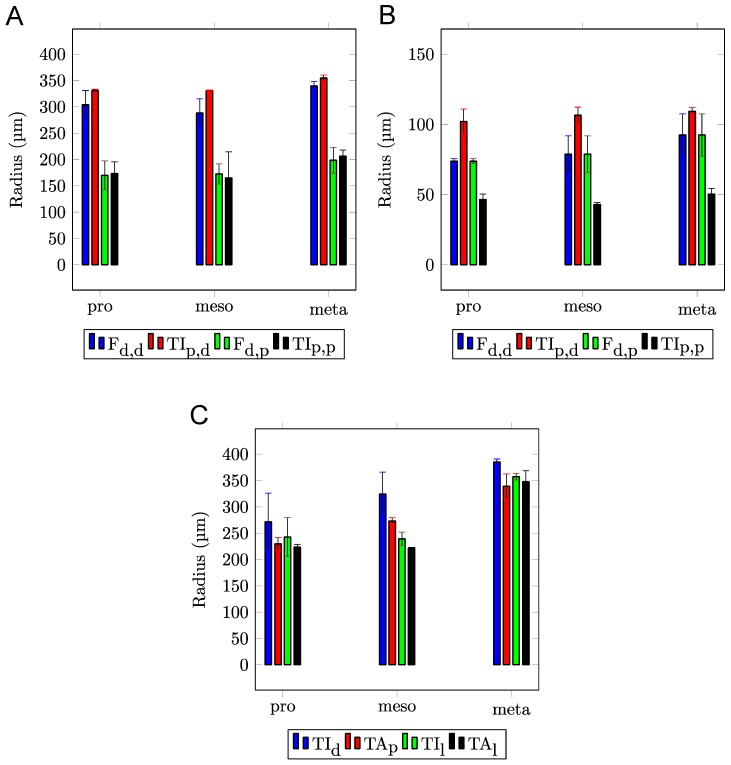
Radii of the contacting surfaces in (**A**,**B**) the femur–tibia and (**C**) the tibia–tarsus leg joints measured by confocal laser scanning microscopy (CLSM). The data for both counterparts of each joint are shown next to each other. Error bars show standard deviations of the means. F_d,p_: the distal end of the femur, the proximal condyle; TI_p,p_: the proximal end of the tibia, the proximal condyle; F_d,d_: the distal end of the femur, the distal condyle; TI_p,d_: the proximal end of the tibia, the distal condyle; TI_d_: the distal end and the condyle of the tibia; TI_l_: the lateral curvature of the distal tibia condyle; TA_p_: the proximal end and the condyle of the tarsus; TA_l_: the lateral curvature of the tarsus condyle; pro: prothoracic leg; meso: mesothoracic leg; meta: metathoracic leg.

Figure 2A–F shows the microscopic images of the articulation surfaces of the examined femur–tibia joint (ft) and the tibial–tarsal joint (tt). To illustrate the contacting conditions of the intact leg joints, Figure 3A–F shows rainbow-colored height profile images. Based on that data, Figure 4A–D shows schematic representations of the joint geometries with the corresponding radii. The concrete measured values of these geometric variables can be found in Supplementary Table S1 for each pair of legs, whereby the contacting radii of the base and counterbody are shown together. All the surfaces under investigation have a convex or concave curvature, which can be described by appropriate radii. Figure 5A–C shows the magnitude of the radii of the individual articulation surfaces next to each other, as they are in contact in the joints. It is clear that a concave contact partner always has a larger curvature than its convex countersurface, so that, for example, the distal condyle of the femur (F) of the prothoracic leg has a radius of *R*_2_ = 303.80 ± 27.33 μm, while the countersurface of the tibia (TI) shows a radius of *R*_6_ = 330.88 ± 2.59 μm. A trend of the increase in dimensions of both radii can be shown from the front, prothoracic leg (pro) to the hind, metathoracic leg (meta) for all joint surfaces.

After the derivation of the primary profile from the measured data, the waviness of the surfaces was examined. A second-order shape deviation can be demonstrated for both the ft and tt joints. In the ft joint, the proximal and distal condyles of the TI are covered by a tooth-like microstructure (Figure 2B,D). The length of an individual tooth is approximately 3 μm and the width at the base is approximately 2 μm. The spatial distribution of the elements seems to be random. The corresponding counterpart is shown in Figure 2A and has a smooth surface. 

A distinctly pronounced microstructure is found in the elliptical basin of the tt joint at the distal end of the TI (Figure 2E). These microstructural elements are specified in more detail in Table 1 by their length, width, and height, whereby it is apparent that no microstructural adaptations are pronounced in the prothoracic leg. The microstructure is more pronounced in *P. marginata* than in the lubber grasshopper (*R. guttata*) [11]. Although the width of individual cuticle outgrowths is approximately 7 μm, the protuberances of *P. marginata* are about twice as long of those in *R. guttata* and significantly higher (3.5–0.5 μm). Qualitatively, these microstructural elements can be characterized by a wedge shape, whose ridge falls from the center to the zero level to the sides. The slightly rising flank of the wedge is always oriented in the distal direction, i.e., in the direction of the joint opening. The individual elements stand out from the basic level of the joint basin. The spatial distribution of the microstructure shows a uniform pattern. In the lateral direction, a continuous gap can be seen per row of protuberances. In the distal direction, the elements are offset from row to row, resulting in a kind of zig-zag groove. The distance between the microstructural elements of the metathoracic leg is Δ_p,d_ = 14.72 ± 2.04 μm in the distal direction, and Δ_l_ = 28.84 ± 4.09 μm in the lateral direction (Table 1). The countersurface of the structured joint basin is the proximal end of the tarsus (TA). This joint ball is shown in Figure 2F and bears a smooth surface in the contact area. 

**Figure 6:**
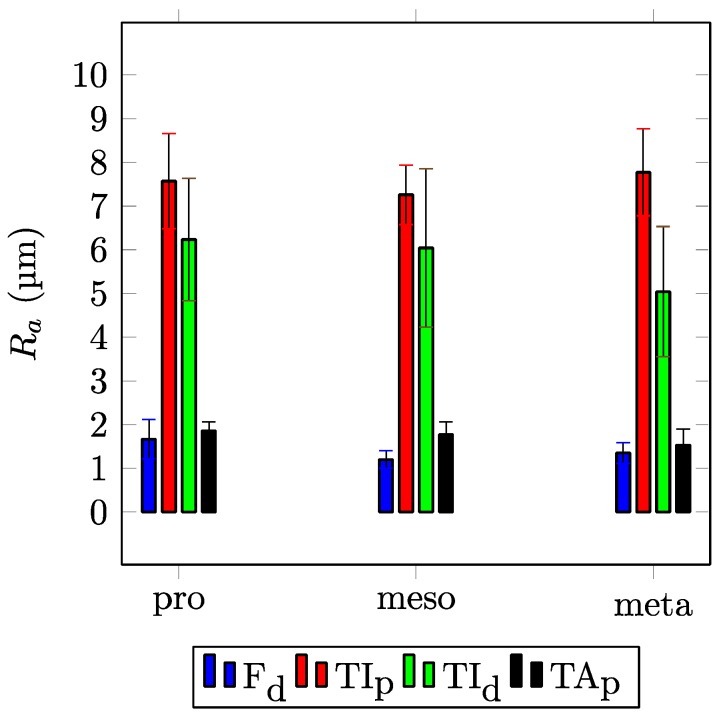
Roughness (*R_a_*) of the condyle in the femur–tibia (F_d_ and TI_p_) and the tibia–tarsus (TI_d_ and TA_p_) leg joints measured by white light interferometry (WLI). Error bars show standard deviations of the means. F_d_: the distal condyle of the femur; TI_p_: the proximal condyle of the tibia; TI_d_: the distal condyle of the tibia; TA_p_: the proximal condyle of the tarsus; pro: prothoracic leg; meso: mesothoracic leg; meta: metathoracic leg.

As a further step, the roughness *R_a_* of the contacting surfaces is determined. Figure 6 shows the measured roughness *R_a_* for the condyles of the ft and tt joints. Contacting surfaces are displayed side by side. It is clear that in both the ft joint (Kruskal–Wallis test by ranks: σ < 0.001) and the tt joint (Kruskal–Wallis test by ranks: σ < 0.001), the roughness of the contacting surfaces is significantly different. For example, for the distal condyle of the F of the metathoracic leg, the value of roughness is *R_a_* = 1.35 ± 0.24 μm and for the corresponding countersurface on the TI, *R_a_* = 7.77 ± 0.99 μm.

The material properties of the contacting surfaces are shown in Table 2. Images of contacting surfaces are displayed below each other. It can also be recognized that in both the ft joint (Kruskal–Wallis test by ranks: σ < 0.03) and the tt joint (Kruskal-Wallis test by ranks: σ < 0.05), the elastic modulus and hardness of surfaces are significantly different. In the tt joint, the surface of TA (*E*_TA,p_ = 2.06 ± 0.95 GPa, *H*_TA,p_ = 0.10 ± 0.02 GPa) is significantly stiffer and harder than the surface of TI (*E*_TI,d_ = 0.47 ± 0.30 GPa, *H*_TI,d_ = 0.04 ± 0.04 GPa).

## 4. Discussion

In a technical or biological tribosystem, when two components are in contact, the microgeometry of both surfaces causes real contact only in discrete microcontacts that deform under the influence of the normal force *F_n_*. Therefore, a distinction must be made between the geometric or nominal contact area *A*_0_ (macroscopic observation) and the significantly smaller real contact area *A_r_*, i.e., the area of the microcontact surfaces (microscopic view) [28]. The true contact surface is of central importance for all tribotechnical systems, since it primarily involves the friction and wear processes [29]. The previous findings of Archard [30] allowed us to estimate the reduction of contact area by protuberances (*A*_2_) in comparison to a nonstructured surface contact (*A*_1_) in the tibia-tarsus joint. Figure 3E shows the condyle of the tibia and can be used for an estimation of both the number and density of initially contacting protuberances (*m* = 4.08 × 10^9^ m^−2^). The coefficients *K*_1_ and *K*_2_, indicating the local curvature of the surface and elastic properties of materials (Figure 3 and Table 2), are calculated with approximations given by Popov [31]: *K*_1_ = 6.2 × 10^−8^ m^2^/N^2/3^ and *K*_2_ = 5.79 × 10^−9^ m^2^/N^8/9^. These boundary conditions are used to calculate the reduction of contact area by protuberances in the tibia–tarsus joint of *P. marginata* (*A*_2_ = *A*_1_/33.2). However, this microscopic contact also entails the risk that unfavorable geometrical dimensions of the contact partners cause an interlocking of the smooth friction partner in the microstructure of the counterface and therefore significantly increase the frictional force [9]. This relationship has already been investigated in the friction of technical structured surfaces [16,32]. Two crucial mechanical interactions between the contacting surfaces can be identified with the effect on the sliding coefficient of friction *μ_k_*: (1) on a nanoscale, by the influence of the real contact area; and (2) on a microscale, by the geometrical and stress-induced interlocking of the samples with the grooves of the structured countersurface. From this, it can be concluded that the dimension of the most friction-optimized biological microstructures must reduce the real contact area of the tribological pair as much as possible without causing mechanical interlocking, both geometrically and contact-mechanically. In technical systems, Sondhauss et al. [32] report that the friction coefficient for a ball microstructure contact pair could be reduced by 50%, if the interlocking could be prevented. Sondhauss et al. [32] and Baum et al. [16,33] show that the friction response is dominated by the geometry of the tribological pair, which is characterized by the macroscopical shape [32,34], as well as the specific geometry of the microstructural elements [35,36,37]. Based on this finding, the previous authors argue that the moderate modification of surface roughness/topology could improve the tribological performance of mesoscale contacts.

**Figure 7:**
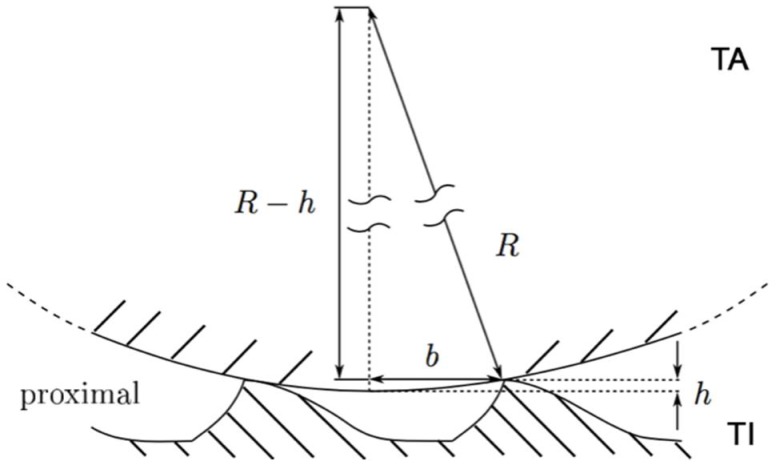
Tibia–tarsus leg joint of *P. marginata*. Schematic illustration of the penetration of the ball cap of the tarsus (TA) into the microstructure of the tibia (TI). *R*: radius of tarsus; *h*: penetration depth; *b*: half-width between two protuberances.

In order to understand possible interlocking of the joint surfaces, the depth *h* was determined, which describes the penetration of the ball cap of the TA into the interstices of the microstructure of the TI (see Figure 7). The purely geometric interaction between the microstructured joint basin of the TI and the smooth spherical TA condyle is analyzed by means of trigonometric functions in connection with the measured geometric variables of the joint surfaces and the microstructure (see Table 1 and Table 2). For the calculation of the geometrical penetration depth using the set of Pythagoras equations (*b*^2^ + (*R* − *h*)^2^ = *R*^2^, cp. Figure 7) for the illustrated right-angled triangle, the dimensions of the two-dimensional distribution of the microstructural elements from Table 1, together with the geometries of the condyles from Supplementary Table S1, are used. The results of the calculation of the penetration depth *h* show the purely geometric portion of the microscopic interlocking for the meso- (*h* = 90.9 nm) and metathoracic (*h* = 79.8 nm) legs of *P. marginata*. The penetration of the TA joint ball between the microstructural elements is clearly below 100 nm and is below 0.1% of the ball radius *R*_12_ or approximately 2–3% of the microstructure height *h_M_*, so that geometrical interlocking in the tt joint is not to be expected.

## 5. Conclusions

The two hypotheses mentioned above can be verified. The real contact area of contacting convex and concave surfaces of the beetle joints is permanently reduced due to the presence of specific curvature, waviness, and microstructure. The functional advantage in friction reduction within insect leg joints can be shown by the specific geometrical shape at different length scales. 

## Figures and Tables

**Table 1:**
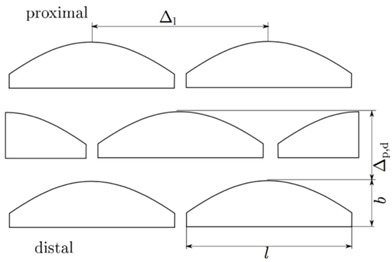
Dimensions of the protuberances in the distal tibia condyle of *P. marginata* measured with white light interferometry (WLI).

	Width (µm)	Length (µm)	Height (µm)	Δ_p,d_ (µm)	Δ_l_ (µm)
Pro	-	-	-	-	-
Meso	6.52 ± 0.60	20.88 ± 1.03	3.21 ± 0.24	14.10 ± 2.45	29.40 ± 10.02
Meta	7.00 ± 0.31	21.43 ± 1.78	3.50 ± 0.39	14.72 ± 2.04	28.84 ± 4.09

pro: prothoracic leg; meso: mesothoracic leg; meta: metathoracic leg; *b*: width of a protuberance; *l*: length of a protuberance; Δ_p,d_: distance between two protuberances in proximal distal direction; Δ_l_: distance between two protuberances in lateral direction.

**Table 2:** Material properties of the cuticle of contacting surfaces in leg joints of *P. marginata*.

	*E* (GPa)	*H* (GPa)
F_d,d_	4.74 ± 2.97	0.10 ± 0.03
TI_p,p_	0.84 ± 0.17	0.03 ± 0.01
TI_p,d_	1.80 ± 0.92	0.05 ± 0.03
TI_d_	0.47 ± 0.30	0.04 ± 0.04
TA_p_	2.06 ± 0.95	0.10 ± 0.02

F_d,d_: the distal end of the femur, the distal condyle; TI_p,p_: the proximal end of the tibia, the proximal condyle; TI_p,d_: the proximal end of the tibia, the distal condyle; TI_d_: the distal end and the condyle of the tibia; TA_p_: the proximal end and the condyle of the tarsus; *E*: Young´s modulus; *H*: hardness.

## Supplementary Materials

The following are available online at http://www.mdpi.com/2313-7673/3/2/12/s1, Table S1: Radii of the contacting surfaces in the femur–tibia and the tibia–tarsus leg joints of *P. marginata*.
