# Supplementary material for: The Topology of the Leg Joints of the Beetle Pachnoda marginata (Scarabaeidae, Cetoniinae) and Its Implication for the Tribological Properties
Source: Biomimetics (Basel). 2018 Jun 8;3(2):12. doi: 10.3390/biomimetics3020012 (PMC6352703; doi:10.3390/biomimetics3020012)
Supplement: Supplementary file 1 [file biomimetics-03-00012-s001.pdf]

Biomimetics **2018**, *3*, 12

Supplementary Materials: The Topology of the Leg Joints of the Beetle *Pachnoda marginata* (Scarabaeidae, Cetoniinae) and Its Implication for the Tribological Properties

Steffen Vagts [1,*], Josef Schlattmann [1], Alexander Kovalev [2] and Stanislav N. Gorb [2]

[1] Department of System Technologies and Engineering Design Methodology, Hamburg University of Technology, Denickestr. 22, D-21079 Hamburg, Germany; j.schlattmann@tuhh.de

[2] Department of Functional Morphology and Biomechanics, Kiel University, Am Botanischen Garten 9, D-24118 Kiel, Germany; akovalev@zoologie.uni-kiel.de (A.K.); sgorb@zoologie.uni-kiel.de (S.N.G.)

* Correspondence: steffen.112@gmx.de; Tel.: +49-40-428784422

Table S1. Radii of the contacting surfaces in the femur–tibia and the tibia–tarsus leg joints of *P. marginata*.

Contact pair	Leg pair	Radius (μm)		Radius (μm)	
$F_{d,p}$ – $TI_{p,p}$	Pro	R_1	169.95 ± 27.38	R_5	173.50 ± 22.34
		R_3	45.95 ± 3.68	R_7	46.5 ± 3.90
	Meso	R_1	172.59 ± 19.06	R_5	165.00 ± 49.50
		R_3	46.93 ± 4.14	R_7	43.00 ± 1.40
	Meta	R_1	198.62 ± 24.54	R_5	206.17 ± 11.92
		R_3	45.28 ± 4.79	R_7	50.30 ± 4.10
$F_{d,d}$ – $TI_{p,d}$	Pro	R_2	303.80 ± 27.33	R_6	330.88 ± 2.59
		R_4	73.80 ± 1.80	R_8	102.00 ± 8.90
	Meso	R_2	288.20 ± 27.01	R_6	331.00 ± 1.10
		R_4	78.80 ± 13.00	R_8	106.50 ± 5.80
	Meta	R_2	339.75 ± 7.93	R_6	354.83 ± 5.67
		R_4	92.50 ± 15.10	R_8	109.30 ± 2.70
TI_d – TA_p	Pro	R_{10}	271.57 ± 54.45	R_{12}	229.86 ± 11.85
	Meso	R_{10}	324.25 ± 41.70	R_{12}	273.25 ± 6.18
	Meta	R_{10}	385.29 ± 5.28	R_{12}	339.43 ± 23.32
TI_l – TA_l	Pro	R_9	242.57 ± 37.14	R_{11}	223.71 ± 5.22
	Meso	R_9	239.25 ± 12.84	R_{11}	221.60 ± 1.52
	Meta	R_9	357.57 ± 6.21	R_{11}	348.00 ± 20.56

$F_{d,p}$: the distal end of the femur, the proximal condyle; $TI_{p,p}$: the proximal end of the tibia, the proximal condyle; $F_{d,d}$: the distal end of the femur, the distal condyle; $TI_{p,d}$: the proximal end of the tibia, the distal condyle; TI_d: the distal end and the condyle of the tibia; TI_l: the lateral curvature of the distal tibia condyle; TA_p: the proximal end and the condyle of the tarsus; TA_l: the lateral curvature of the tarsus condyle; pro: prothoracic leg; meso: mesothoracic leg; meta: metathoracic leg; $R_{1–12}$: radii of contacting joint surfaces.
